# Tideglusib accelerates bone–tendon interface healing and improves mechanical strength in a rabbit rotator cuff tear model: an experimental study

**DOI:** 10.1186/s13018-026-06717-3

**Published:** 2026-02-14

**Authors:** Zeynel Can Ocaklar, Murat Serkant Ünal, A. Çağdaş Yörükoğlu

**Affiliations:** 1 Orthopedics and Traumatology Department, Pamukkale University Medical Faculty, 20070 Pamukkale, Denizli, Turkey; 2 Department of Medical Histology and Embriyology , Pamukkale University Faculty of Medicine, 20070 Pamukkale, Denizli, Turkey

**Keywords:** Tideglusib, Rotator cuff, Bone-tendon interface healing

## Abstract

**Background:**

The present study aimed to investigate the effects of the glycogen synthase kinase-3 (GSK-3) inhibitor tideglusib on bone-tendon interface healing in a rabbit model of rotator cuff injury, based on biomechanical and histological assessments.

**Methods:**

Fourteen New Zealand rabbits underwent supraspinatus tendon detachment to establish a chronic rotator cuff tear model. After six weeks, surgical repair was performed. In the right shoulders, tideglusib was administered at the bone-tendon junction prior to performing the primary repair (drug group), whereas the left shoulders underwent primary repair without biological augmentation (control group). Seven animals were included in the group subjected to biomechanical tension testing, and six for histological evaluation.

**Results:**

Biomechanical evaluation demonstrated that the tideglusib group showed significantly higher load-to-failure values compared with the control group the control group (*p* < 0.05), whereas elongation at failure showed no statistically significant difference. Histological scoring demonstrated significantly improved cellular organization and tissue healing in the tideglusib group (*p* < 0.05).

**Conclusion:**

Local application of tideglusib positively enhances tendon–bone healing both biomechanically and histologically. Further studies are warranted to explore its potential clinical applications.

## Introduction

Rotator cuff tears are recognized as one of the most common causes of shoulder pain and upper extremity dysfunction in the adult population, and they represent a significant clinical problem, particularly due to their increasing prevalence in the elderly [1]. These tears mostly show a heterogeneous spectrum associated with degenerative processes and can present with clinical pictures ranging from asymptomatic cases to significant loss of function [2]. Despite improvements in operative reconstruction methods, high re-tear rates remain an important issue in the literature [3–6]. A primary factor contributing to these failures is insufficient histological healing at the bone-tendon interface [7–9].

Various strategies have been investigated to support the regenerative process at the bone–tendon interface, including modifications in suture quantity, fixation methods, and the use of biological agents [10]. Among these, biological augmentation has gained increasing attention. Osteoblastic activity and neovascularization originating from the underlying bone have been shown to play a role in tissue regeneration, while the tendon stump itself has limited vascular supply [8].

The success of tendon-bone interface healing after rotator cuff repair depends on the regeneration of the fibrocartilage layer, a key component of the natural enthesis structure. Experimental studies have shown that the Wnt/β-catenin signaling pathway is associated with fibrocartilage formation at the tendon-bone interface [11, 12]. Tideglusib, a small molecule agent that is a glycogen synthase kinase-3 (GSK-3) inhibitor, has been associated with regenerative tissue responses in bone and dentin models by modulating this signaling pathway [13, 14]. Therefore, the use of tideglusib was preferred in this study to affect the biological mechanism targeting fibrocartilage-mediated tendon-bone healing.

The initial development of Tideglusib focused on its potential in treating Alzheimer’s disease and progressive supranuclear palsy [15]. Recent studies have shown that it promotes reparative dentinogenesis and osteoblastic proliferation through mechanisms involving the Wnt/β-catenin signaling pathway [16–18]. Bone and dentin, share a similar embryological origin and extracellular matrix composition, it has been suggested that tideglusib may have comparable effects on bone tissue. Indeed, previous experimental studies demonstrated that tideglusib enhanced new bone formation and mineralization in fracture and bone defect models [11, 19].

To our knowledge, no previous study has investigated the effects of tideglusib on bone–tendon interface healing. Therefore, this experimental study aimed to evaluate the biomechanical and histological effects of local tideglusib application in a rabbit model of chronic rotator cuff tear.

## Materials and methods

This experimental study was approved by the Pamukkale University Animal Experiments Local Ethics Committee (PAUHADYEK-2020/15) at the 2020/03 meeting held on June 26, 2020. The animals used in this study were obtained from the Experimental Animal Research Center of Pamukkale University. All animal experiments were conducted following institutional guidelines for the care and use of laboratory animals, in compliance with the principles of the Declaration of Helsinki. Fourteen adult white New Zealand rabbits (mean weight 3–3, 5 kg) were used. Animals were maintained under standard laboratory conditions, and anesthesia was provided using ketamine (35 mg/kg) and xylazine (5 mg/kg).

An experimental model of chronic rotator cuff tear was developed by detaching the supraspinatus tendon from its humeral insertion and placing a Penrose drain at the tendon ends. After a 6-week waiting period, a second surgery was performed.

**Fig. 1:**
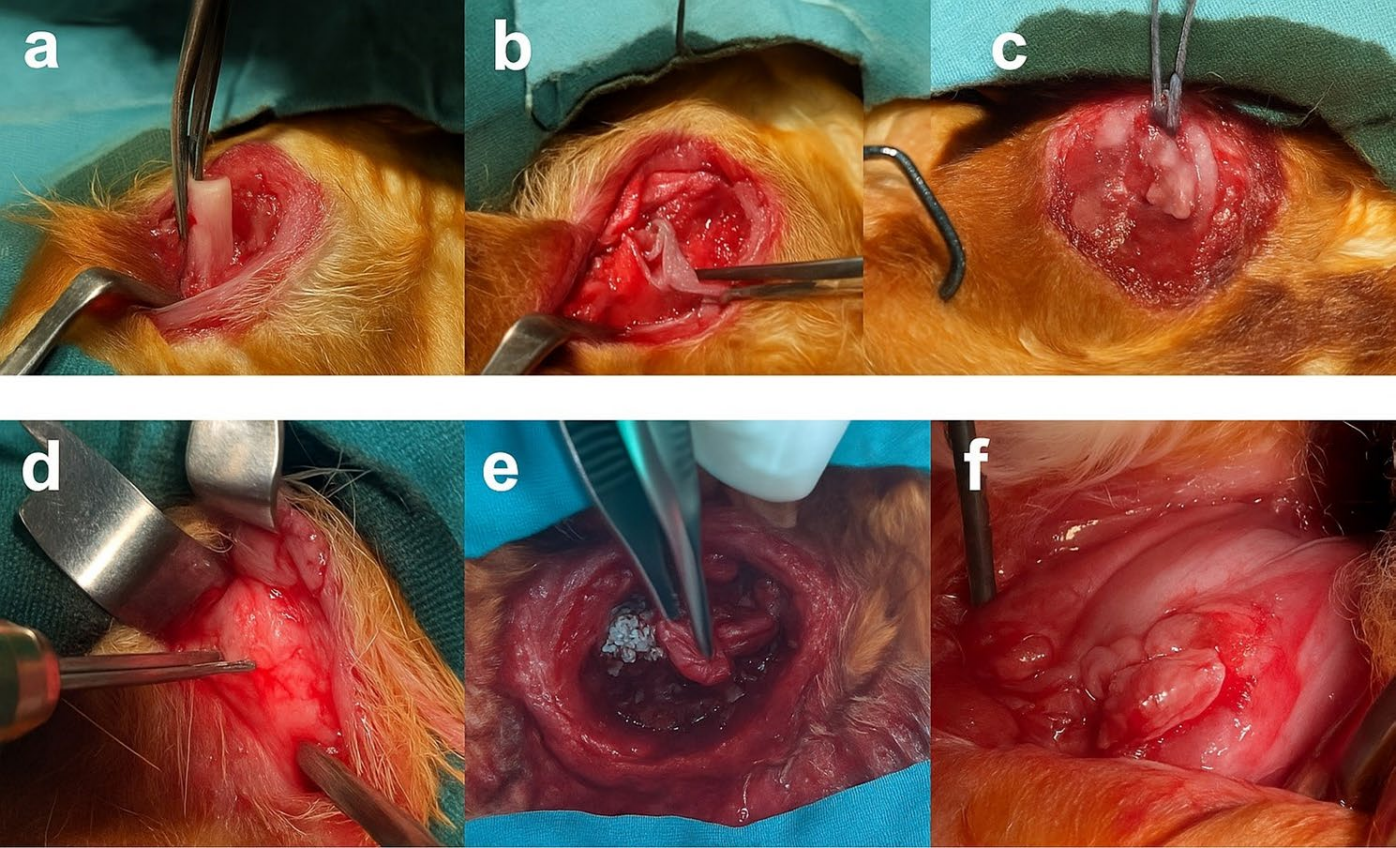
Surgical procedure: **a**–**c**) Detachment of the supraspinatus tendon and preparation with Penrose drain, **d** drilling at tendon insertion, **e** tideglusib application and tendon approximation, **f** tendon fixation with sutures

During the second surgery, a bone tunnel was created in the humerus of the right shoulder, and tideglusib was locally applied to the bone–tendon interface before primary repair with 4/0 Vicryl sutures (drug group). In the left shoulder, primary repair was performed without biological augmentation (control group). One rabbit died postoperatively and excluded from the analyses. Following the experimental procedures, all test animals were humanely euthanized under general anesthesia. Anesthesia was administered using intraperitoneal injections of ketamine (50 mg/kg) and xylazine (10 mg/kg). After confirmation of loss of reflexes and loss of consciousness, euthanasia was performed by administering a high dose of anesthetic overdose, in accordance with institutional and international guidelines for the humane use of laboratory animals. Therefore, seven rabbits (right and left shoulders) were included for biomechanical evaluation, and six rabbits (right and left shoulders) were included for histological assessment (Fig. 1).

**Fig. 2:**
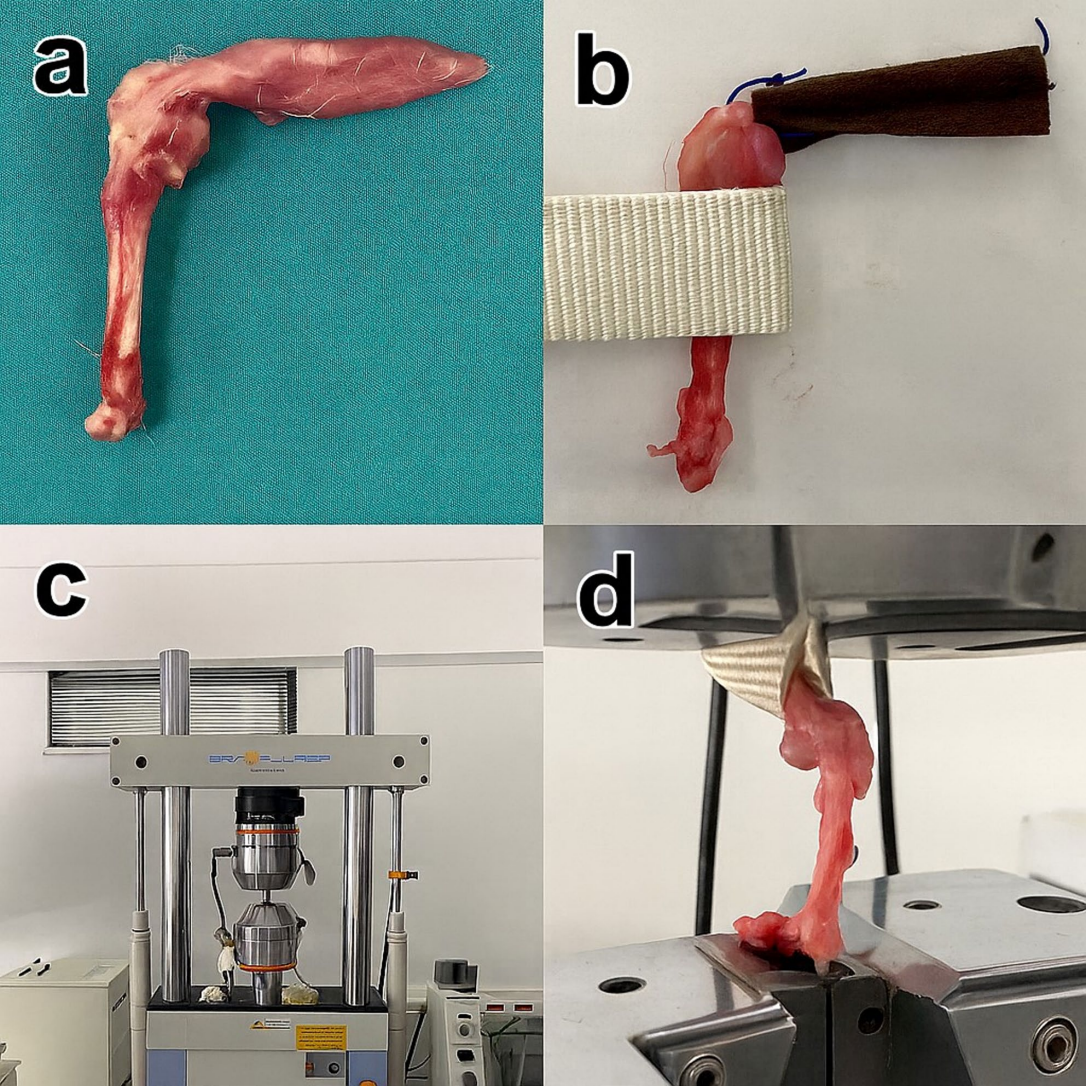
Biomechanical testing: **a** Prepared tendon–bone unit, **b** SHIMADZU EHF-EV200k2-040-0 A tensile testing device, **c** and **d** representative failure specimens after testing

For biomechanical testing, tendon–bone units were mounted on a SHIMADZU EHF-EV200k2-040–0 A tensile testing device. Load–elongation curves were recorded, and the load to failure (N) and elongation at failure (mm) were measured (Fig. 2).

Histological specimens were fixed in 10% formalin, embedded in paraffin, and stained with Hematoxylin–Eosin (H&E), Masson Trichrome, Alcian Blue, and Collagen Type II immunohistochemical staining. Bone–tendon interface (TBI) specimens were stained according to standard histological protocols and evaluated blindly by two independent histologists. The bone–tendon interface healing process was scored between 1 and 4 according to the following criteria: (A) cell density at the tendon–bone interface, (B) collagen type II content, (C) glycosaminoglycan (GAG) content, (D) collagen organization, and (E) chondrocyte organization described by Nourissat [20].

### Statistical analysis

Statistical analyses were conducted using SPSS version 22.0 (IBM, USA). Continuous variables exhibiting normal distribution were presented as mean ± SD, and statistical comparisons were made using the Student’s t-test. Variables that did not follow a normal distribution were reported as median (minimum–maximum), and comparisons were performed using the Mann–Whitney U test. A *p*-value of < 0.05 was considered statistically significant.

## Results

One rabbit died during the study, leaving 13 rabbits used for evaluation. Biomechanical tensile testing revealed that the average load to failure in the tideglusib-treated shoulders was 101.7 ± 18.3 N, whereas the control group exhibited 66.9 ± 11.2 N. A statistically significant difference was observed between the groups (*P* < 0.05), indicating that tideglusib enhanced the mechanical strength of the bone–tendon interface (Table 1). No significant differences were observed in elongation at failure between the groups; the mean elongation was 3.98 ± 0.7 mm for the tideglusib group and 3.14 ± 0.5 mm for the control group (*P* > 0.05) (Table 2).

**Table 1:** Load to failure results in Biomechanical testing

Group	Mean ± SD (*N*)	Minimum (*N*)	Maximum (*N*)	*p*-value
Tideglusib	101.7 ± 18.3	73.8	126.6	< 0.05
Control	66.9 ± 11.2	55.3	84.7	–

SD, standard deviationA p-value < 0.05 indicates a statistically significant difference

**Table 2:** Elongation at failure measurements

Group	Mean ± SD (mm)	Minimum (mm)	Maximum (mm)	*p*-value
Tideglusib	3.98 ± 0.70	3.2	4.8	> 0.05
Control	3.14 ± 0.50	2.6	3.9	–

SD, standard deviationNo statistically significant difference was observed between groups

**Fig. 3:**
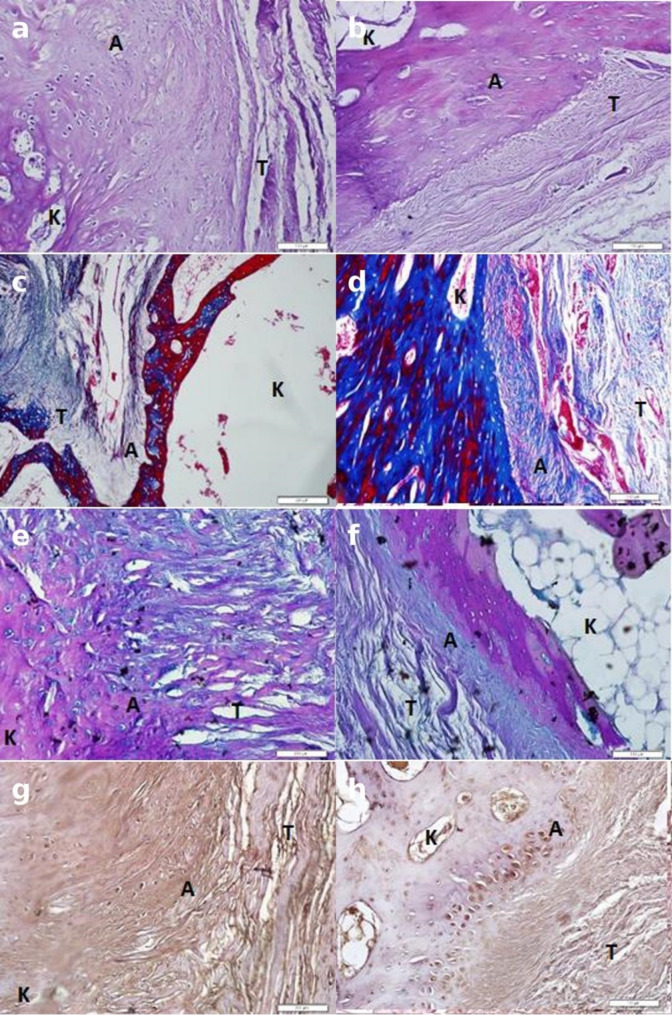
Histological and immunohistochemical evaluation (20×): **a** and **b** H&E staining, **c** and **d** Masson Trichrome staining, **e** and **f** Alcian Blue staining, **g** and **h** Collagen Type II immunostaining. Tideglusib-treated samples show improved fibrocartilaginous transition zone, more organized collagen fibers, and stronger Collagen II expression compared with control samples

Histological evaluation revealed irregular collagen fibers, low cellularity, and limited fibrocartilaginous tissue formation in the control group. In contrast, the Tideglusib-treated group exhibited a well-organized fibrocartilaginous transition zone, a homogeneous distribution of chondrocyte-like cells, and improved collagen alignment. Immunohistochemical analysis demonstrated stronger Collagen Type II staining in the Tideglusib group (Fig. 3).

According to the Nourissat histological scoring system, Tideglusib-treated specimens demonstrated significantly higher scores for cell density, collagen type II, and glycosaminoglycan (GAG) content (*P* < 0.05), indicating enhanced bone–tendon interface healing compared with controls. No significant difference was detected in collagen organization (*P* = 1.000). Although chondrocyte organization did not reach statistical significance (*P* = 0.065), columnar alignment of chondrocytes was more prominent in the Tideglusib group (Table 3).

**Table 3:** Histological scoring results based on the nourissat system

Parameter	Tideglusib Group	Control group	*p*-value
A. Cell density	3.67 ± 0.52 / 4 (3–4)	2.50 ± 0.55 / 2.5 (2–3)	0.015*
B. Collagen type II	3.67 ± 0.52 / 4 (3–4)	2.67 ± 0.52 / 3 (2–3)	0.026*
C. GAG content	3.67 ± 0.52 / 4 (3–4)	2.67 ± 0.52 / 3 (2–3)	0.026*
D. Collagen organization	3.67 ± 0.52 / 4 (3–4)	3.67 ± 0.52 / 4 (3–4)	1.000
E. Chondrocyte organization	3.50 ± 0.55 / 3.5 (3–4)	2.67 ± 0.52 / 3 (2–3)	0.065

GAG, glycosaminoglycanValues presented as mean ± SD and median (range). *Indicates *p* < 0.05.

## Discussion

This study evaluated the biomechanical and histological effects of tideglusib in a rabbit model of chronic rotator cuff tear. Biomechanical testing revealed a significantly higher load to failure in the tideglusib group, whereas no significant differences were observed in elongation. Histological analysis demonstrated increased cellularity, GAG content, and Collagen Type II expression in the tideglusib group compared to controls, although collagen and chondrocyte organization, while clinically improved, did not reach statistical significance.

Rotator cuff tears are the common clinical problem in orthopedics, and the aim of surgical repair is to restore the bone–tendon interface as anatomically as possible. Despite early diagnosis and repair, physiological healing of the bone–tendon interface is often incomplete [4–6]. Therefore, strategies to enhance healing, including biological augmentation, are critical [7, 8]. In this study, tideglusib, known for its osteoblastic activity, was applied to support the underlying bone.

Tideglusib, originally developed as a therapeutic agent for progressive supranuclear palsy and Alzheimer’s disease, inhibits GSK-3 and has been reported to stimulate the Wnt/β-catenin signaling cascade, promoting differentiation of osteoblasts and subsequent bone formation [15, 16, 18]. Previous experimental studies have reported enhanced bone formation and mineralization with local tideglusib application in fracture and bone defect models [11, 19]. To the best of our knowledge, its effects on the bone–tendon interface have not been thoroughly investigated, and the present study aimed to contribute to this limited body of evidence.

The Wnt/β-catenin pathway has also been implicated as an essential mediator of bone–tendon interface healing in studies involving other therapeutic agents [12–14, 21]. We hypothesize that tideglusib accelerates healing by activating tendon-derived stem cells via this pathway, consistent with our observed increase in biomechanical strength. Furthermore, the use of a chronic tear model and immunohistochemical evaluation of collagen subtypes enhances the reliability of our findings [22, 23]. In this study, the evaluations regarding the Wnt/β-catenin signaling pathway were addressed based on information obtained from the existing experimental literature, without direct analysis at the molecular or cellular level. The literature reports that the Wnt/β-catenin signaling pathway does not exhibit a uniform biological effect in tendon and enthesis tissues; it can elicit different cellular and tissue responses depending on factors such as the activation level, application time, and dose exposure [12–19]. In this context, considering experimental studies, it is thought that modulation of the Wnt/β-catenin signaling pathway may affect healing processes at the tendon-bone interface [12, 16]. Within this framework, the biomechanical and histological findings obtained in this study suggest that early fibrocartilage-like healing processes at the tendon-bone interface are supported, consistent with increased cellular organization, glycosaminoglycan content, and Collagen Type II expression. However, further molecular and cellular studies are needed to clearly elucidate the precise mechanisms of these effects and their roles in actual enthesis regeneration.

Histological evaluation revealed increases in cellular density, glycosaminoglycan content, and collagen type II expression, suggesting a fibrocartilage-like healing response at the tendon-bone interface. However, the lack of a significant difference in collagen fiber organization between groups and the statistically insignificant improvement in chondrocyte organization indicate that these findings do not reflect a fully mature enthesis regeneration. Therefore, the current histological findings point to an early or accelerated phase of tendon-bone healing rather than comprehensive interface regeneration. Considering that the structural and functional maturation of the enthesis is a time-progressive process, studies with longer follow-up periods are needed to demonstrate the continuity and organizational maturation of these healing patterns.

This study has some methodological limitations. First, the sample size used in the experimental design is limited, and dose optimization for tideglusib and the release kinetics of the drug at the tendon-bone interface were not evaluated in this study. Furthermore, the six-week follow-up period does not allow for definitive conclusions about the long-term durability of the biomechanical gains obtained and their persistence on the mature enthesis structure.

From a translational perspective, issues such as the optimal dose, delivery systems, and safety profile of tideglusib for local application need to be investigated in detail before clinical use. Therefore, rather than providing results that can be directly transferred to clinical practice, the current findings are experimental evidence demonstrating that tideglusib is a potential biological agent that can support tendon-bone healing. Future studies with larger sample sizes, different doses and application strategies, and longer follow-up periods will more clearly demonstrate the clinical applicability and safety of this agent.

## Conclusion

This experimental study demonstrated that local tideglusib application enhances bone–tendon interface healing in a rabbit rotator cuff tear model. Biomechanical evaluation showed a significantly higher load to failure in the tideglusib group, whereas elongation did not differ significantly between groups. In the tideglusib group, histological analysis demonstrated higher cellularity, greater GAG deposition, and increased Collagen Type II expression compared with the control group.

These results suggest that tideglusib positively affects bone–tendon interface healing both biomechanically and histologically. Further studies are warranted to optimize dosing, application protocols, and to investigate long-term effects in both experimental and clinical settings.

## Data Availability

The datasets generated and/or analysed during the current study are available from the corresponding author on reasonable request.
